# Investigation of the Gamma-ray Shielding Performance of CuO-CdO-Bi_2_O_3_ Bentonite Ceramics

**DOI:** 10.3390/ma15155310

**Published:** 2022-08-02

**Authors:** Hanan Al-Ghamdi, Mohamed Elsafi, Aljawhara H. Almuqrin, Sabina Yasmin, M. I. Sayyed

**Affiliations:** 1 Department of Physics, College of Science, Princess Nourah Bint Abdulrahman University, P.O. Box 84428, Riyadh 11671, Saudi Arabia; hmalghmdi@pnu.edu.sa (H.A.-G.); ahalmoqren@pnu.edu.sa (A.H.A.); 2 Physics Department, Faculty of Science, Alexandria University, Alexandria 21511, Egypt; 3 Department of Physics, Chittagong University of Engineering and Technology, Chattogram 4349, Bangladesh; sabinayasmin309@gmail.com; 4 Department of Physics, Faculty of Science, Isra University, Amman 11622, Jordan

**Keywords:** bentonite, ceramics, Bi_2_O_3_, Energy Dispersive X-ray, HPGe detector, radiation shielding

## Abstract

The purpose of this research is to identify the radiation shielding capability of ceramics adding CuO, CdO, and Bi_2_O_3_ with diverse wt (%). The chemical compositions of the raw ceramics were documented through Energy Dispersive X-ray “EDX” techniques. For aesthetic appeal and solidification, CuO has been chosen to be added to ceramic. Moreover, in the interest of increasing the radiation shielding ability, the high atomic number and density of both CdO and Bi_2_O_3_ were suggested for the raw ceramics. To obtain the morphological features of the prepared ceramic samples, a Scanning Electron Microscope, or SEM, was utilized. To verify the experimental results, the MCA value obtained from the Phy-X software was compared to the experimental value collected from the HPGe detector. At energies 0.06 MeV, 0.662 MeV, 1.173 MeV, and 1.333 MeV the linear and mass attenuation coefficients of the prepared ceramics have been measured using a high purity germanium “HPGe” detector as well as three different point sources. Moreover, the relationship between ln(I) and the thickness of the ceramics has been presented here, and the comparison between the LAC of the prepared ceramics with other materials has also been displayed. Bentonite ceramic containing CuO (15 mol %)-CdO (15 mol %)-Bi_2_O_3_ (20 mol %) with density 3.6 showed the lowest HVL, MFP, and TVL at all studied energies, yet pure Bentonite ceramic containing only CuO (50 mol %), having density 3.4, presented the greatest values. Hence, it can be concluded that the addition of CdO and Bi_2_O_3_ enhances the radiation shielding ability.

## 1. Introduction

Gamma photons have extremely high frequency and energy, and thus have a high penetrating ability through matter. These photons (especially those which carry high energy) can easily penetrate human tissues and organs, and thus they are considered very dangerous for both humans and the environment. It is well known that gamma photons have negative effects on the human body and can cause damage to DNA and cells. In any nuclear or medical facility, a set of precautions must be followed, and one of the most important precautions is the use of radiation-protective materials [1,2,3,4]. Historically, lead and concretes have been extensively applied in radiation shielding applications thanks to their effective attenuation ability, high density, availability, inexpensive price, elevated attenuation factors, and low maintenance [5]. Nevertheless, recent efforts in the radiation protection field demonstrated that they have some drawbacks such as health hazards due to the toxicity of the lead, poor mechanical properties, and heavy weight. Based on these drawbacks and other disadvantages of these traditional materials, more efforts are required to investigate the shielding effectiveness of other alternative materials. In recent years, different novel materials include alloys, glasses, polymers, rocks, ceramics, and composites [6,7,8,9,10]. Among these materials, ceramics are promising materials for radiation shielding thanks to their high thermal durability, good mechanical features, high corrosion resistance, high effective shielding ability, and thermal conductivity. Due to these interesting features of ceramics, they are considered a good candidate for radiation protection. Previous works demonstrated that the attenuation features of ceramics may outperform the properties of other materials, so it is significant to test the radiation attenuation characteristics of newly developed ceramics [11,12]. Bentonites contain large amounts of montmorillonite clay minerals, produced by the alteration of volcanic ash in marine environments. Bentonite consists of extremely small particles, most less than one micron. Bentonites are on the order of ten times finer than ball clays. There are two general classes of bentonites: the CaO-rich bentonites that do not swell much in water, and the CaO-poor, Na_2_O-rich bentonites that swell considerably. Bentonite is the most plastic material used in ceramics. Bentonites are used to add plasticity to clay bodies and help to suspend glaze ingredients [13].

Irradiation energy, temperatures, and pre-existing defects create an effect by adding numerous samples to the Al_2_O_3_ [14]. Adding TiO_2_, Zinc oxide ZnO, CuO, and CdO metals into the thick film varies electrical properties through irradiation. Enhancement of the radiation dose amplifies the leakage current. The CuO/Si and CdO/Si diodes increase current according to dose [15]. Oxygen vacancies are the foremost defects of β-Ga_2_O_3_: Mg^2+^ single crystals where Ga^3+^ ions are substituted by Mg^2+^ ions in lattice positions. Mg^2+^ ion doping on the β-Ga_2_O_3_ single crystals provides photoconductivity as oxygen vacancies are produced from the trap centers by liberating nonequilibrium electrons [16]. The over-stoichiometric cadmium atoms on the Cadmium iodide (CdI_2_) layered crystals form clusters [17]. Briefly, we summarized the previous efforts of the researchers that focused on the radiation attenuation study for different kinds of ceramics. Asal et al. studied natural bentonite clay-made ceramic materials for gamma radiation shielding. The shielding ability of the studied samples has been measured using ^251^Am, ^57^Co, ^137^Cs, ^60^Co, and ^88^Y point sources. The value of LAC varied from 0.479–1.06 cm^−1^, and the value of mass attenuation coefficients lay between 0.238 and 0.443 cm^2^/g, according to their thicknesses [18]. Oto et. al. researched the shielding capability of standard ceramic- and Molybdenum (Mo)- doped ceramics against gamma radiation. Ceramic containing 30% Mo showed higher mass attenuation coefficients and effective atomic numbers; however, lower mean free paths compared with other studied ceramics. The amount of Mo increased the gamma shielding ability of the studied ceramics, yet no significant variance was displayed against fast neutrons [19]. Usta et al. researched the structural, morphological, and radiation shielding properties of the hexagonal boron nitride ceramic material doped with Ni-Co–B for diverse current densities. The radiation shielding ability of the studied samples was reduced with an increase of current density. For a current density of 100 mA/cm^2^, the amalgamation level of hexagonal boron nitride ceramic materials was 0.68 [20]. Sayyed et al. reported the radiation shielding characteristics of bi-ferric ceramics with carbon nanotube (CNTs) contamination. The studied C1 ceramic sample presented a tetragonal structure. A discrepancy of the lattice constant, as well as shrinkage in the cell unit volume V, was displayed through the addition of the CNTs into bi-ferric ceramic [21]. Hannachi et al. showed that the structural, optical, and radiation shielding properties of ferroelectric BaTiO_3_ ceramics are predisposed by different oxide doping. For ZnO doping, BaTiO_3_ sustained the tetragonal structure; nevertheless, WO_3_ and SiO_2_ doping presented a cubic structure with few impurities. SiO_2_-doped BaTiO_3_ showed an extensive bandgap energy, comparable to all other studied ceramics. The values of linear attenuation coefficients (μ) of BaTiO_3_ increased progressively through the contamination of ZnO or WO_3_ metal oxides at energies 0.244, 0.662, and 1.332 MeV, correspondingly. Likewise, the accumulation of ZnO and WO_3_ improved the half-value thickness (Δ0.5) a little with 1.142% and 4.532%, respectively, yet SiO_2_ contamination increases the Δ0.5 value to 9.16% [22]. Temir et. al. studied the radiation shielding efficacy of Bi_2_O_3_-TeO_2_-WO_3_ ceramics. With an increase of thickness from 0.3–0.4 mm, a high-pitched increase has been found up to 25–50% of radiation; however, the shielding efficiency did not exceed 3–9% for the thickness of 0.1–0.2 mm [23].

The purpose of this research is to identify the radiation shielding capability of ceramics adding CuO, CdO, and Bi_2_O_3_ with diverse wt (%).The gamma ray attenuation coefficients for new types of ceramics were calculated using experimental and theoretical methods.

## 2. Materials and Methods

### 2.1. Sample Preparation

**Figure 1:**
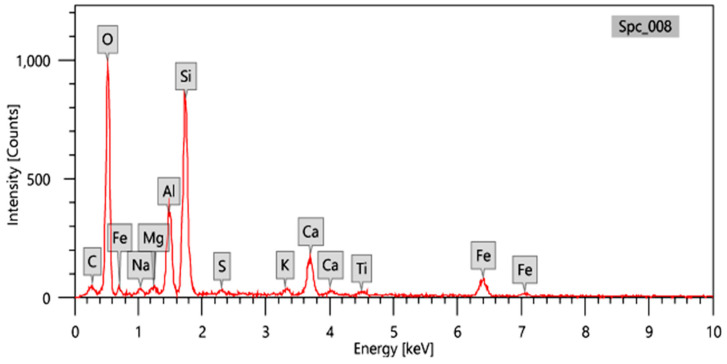
EDX analysis of Bentonite clay.

In this work, the raw materials of these ceramic samples were Bentonite clay, Copper oxide (CuO), Cadmium oxide (CdO), and Bismuth oxide (Bi_2_O_3_). Each material was weighed using a sensitive balance, and the matrix was bentonite clay as a major component in the manufacture of ceramics. The bentonite clay was collected from a quarry in the Fayoum region in Egypt in the form of a powder, sieved with a 60 μm sieve, and analyzed using EDX analysis to identify the components and their proportions (where a quantity of powder was placed in the device under the influence of a voltage of 20 kV and a magnification factor of 500). Figure 1 shows the EDX analysis of bentonite and Table 1 shows the percentage of the elements present in the used bentonite.

CuO was chosen to maintain the hardness of the ceramic and give it an attractive look at the same time [24]. Both CdO and Bi_2_O_3_ were selected for their high atomic number and density, and they have high absorption points at different energies, and thus give higher results in attenuating the radiation falling on the material, where Bi_2_O_3_ is a good attenuator for gamma rays while CdO is a perfect attenuator for neutrons [25,26,27]. The materials were mixed in the same percentages as tabulated in Table 2, then water was added and stirred well until it became a paste and then poured into cylindrical molds. The samples were left to dry for a week, after which they were inserted into an electric oven, and the temperature was gradually raised to 800 °C [28]. The density was measured, where the masses were weighed with a sensitive balance, and the volume was equal ($\pi r^{2}.x$), where x and r represents the thickness and radius of the sample.

### 2.2. SEM Test

SEM of the JEOL Model in the Electron Microscope Unit, Faculty of Science, Alexandria University was used to image the prepared ceramic samples to discuss the morphological properties of these samples [29].

### 2.3. Photon Attenuation Test

**Figure 2:**
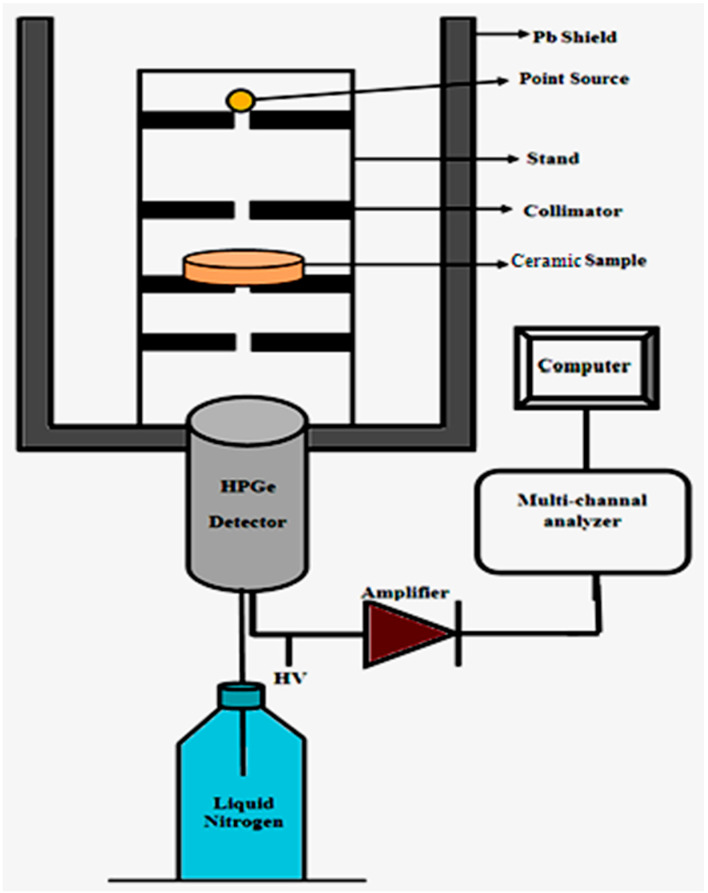
The geometry of the experimental setup.

A high purity germanium “HPGe” detector model CS20-A31CL and three different point sources, Am-241, Cs-137, and Co-60, were used in the Institute of Graduate Studies and Research, Alexandria University, Egypt, to determine the attenuation parameters of the present ceramics. The geometry used in this study is illustrated in Figure 2. It was first calibrated to find out the appropriate place between the source and the detector for the sample to be measured using samples with a known attenuation coefficient. Electrons resulting from the interaction of photons with the detector accumulate to give different peaks. Wach peak corresponds to a different energy; these peaks depend on the intensity of the incident photon, the more the intensity of the photon increases the area of this peak and vice versa [30,31,32].

In this work, the area under the peak was calculated within and without the ceramic sample at the same time using Genie 2000 software. From these areas, the mass attenuation coefficient (MAC) can be calculated by the following formula [33]:

(1) $$MAC=\frac{1}{t\times \rho }\;ln\frac{A_{0}}{A}$$

where, $A_{0}\;$ and A represent the measured area without and within the ceramic sample, respectively; t (cm)  and $\rho \;(g/{\mathrm{cm}}^{3}$) represent the thickness and density of the measured sample, respectively. The experimental results were compared with the results obtained from Phy-X software, where the MAC was calculated with a wide range of energies. The LAC is an essential factor for measuring the shielding materials and can be measured or calculated depending on the MAC calculations by the next equation [34]:

(2) LAC=MAC×ρ

The half and tenth value layers (HVL and TVL) are calculated by the following equations [35,36,37]:

(3) $$HVL=\frac{In\;\left(2\right)}{LAC}\;TVL=\frac{In\;\left(10\right)}{LAC}$$

## 3. Results and Discussion

**Figure 3:**
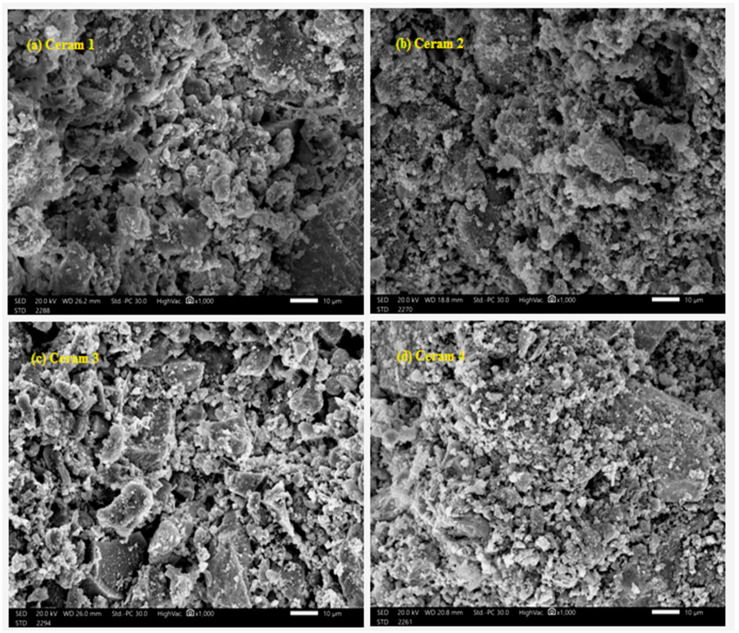
SEM images of Ceramic samples, (**a**) Ceram 1, (**b**) Ceram 2, (**c**) Ceram 3 and (**d**) Ceram 4.

In Figure 3, SEM images of the prepared ceramic samples show that the voids in the bentonite decrease with the increase in the proportion of heavy oxides, and this indicates the improvement of the morphological properties, which in turn leads to an increase in the rate of photon absorption when they fall on this material, and thus makes it more efficient shielding.

**Figure 4:**
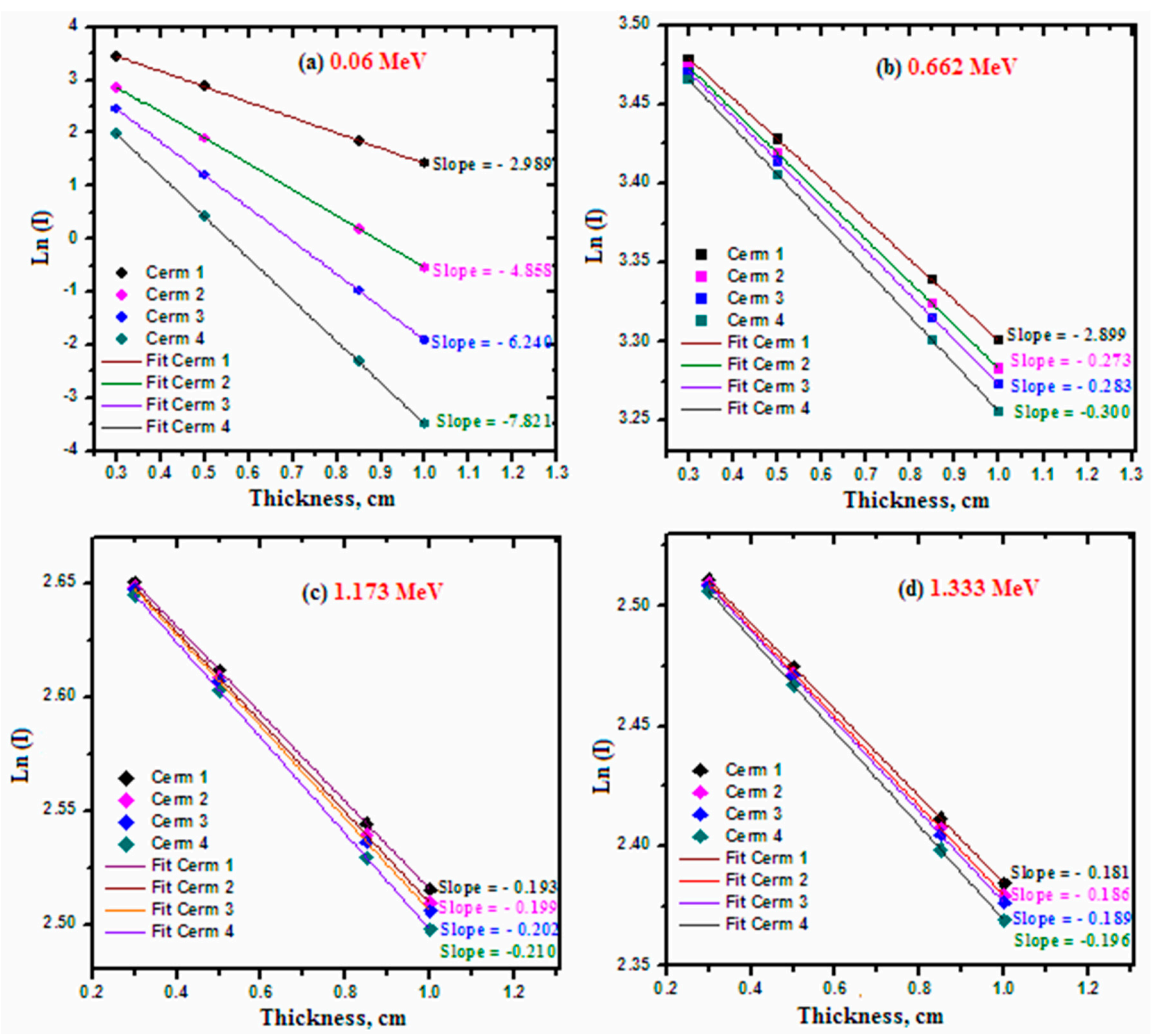
Relation between ln(I) and the thickness of the ceramic samples.

**Figure 5:**
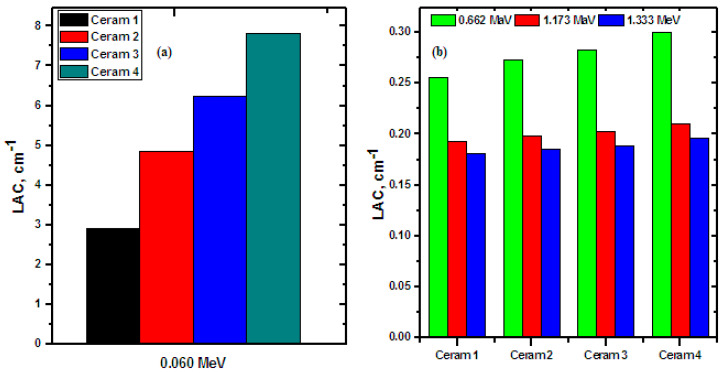
Linear attenuation coefficients for the prepared ceramic samples, (**a**) at 0.060 MeV and (**b**) at 0.662, 1.173 and 1.333 MeV.

In Figure 4, the relationship between ln(I) and the thickness of the ceramic sample was graphed at four chosen energies, which vary from the low to the high energy range; more specifically. The results of these figures were used to calculate the LAC of the samples, as LAC = (ln(I_0_) − ln(I))/thickness, where the y-intercept of the graphs represents ln(I_0_) and the slope of each set of data points is the LAC value at each energy. The figure demonstrates that long(I) decreases as the energy increases. At 0.06 MeV, the slope of Ceramic 1 is −2.989 while the slope of Ceram 4 is 7.821, which shows that the slope of the ceramic samples increases in magnitude as CdO and Bi_2_O_3_ are added to the samples at the expense of CuO. In other words, increasing the CdO and Bi_2_O_3_ content of the studied ceramic samples increases their LAC values. This same trend is also observed at all other tested energies but is most apparent at lower energies. The LAC values of the ceramic samples were plotted in Figure 5 to illustrate that the LAC values of the ceramic samples follow the order: Ceram 4 > Ceram 3 > Ceram 2 > Ceram 1 at all four tested energies. This is because Ceramic 4 has a higher amount of Bi_2_O_3_ than the other ceramic samples, as well as higher density. In other words, as we move from Ceramic 1 to Ceramic 4, the concentration of Bi_2_O_3_ increases, and the concentration of CuO is decreased, thus the density of the ceramics shows an increasing trend with the concentration of Bi_2_O_3_, and it is known that the LAC is directly related to the density of the shield.

**Figure 6:**
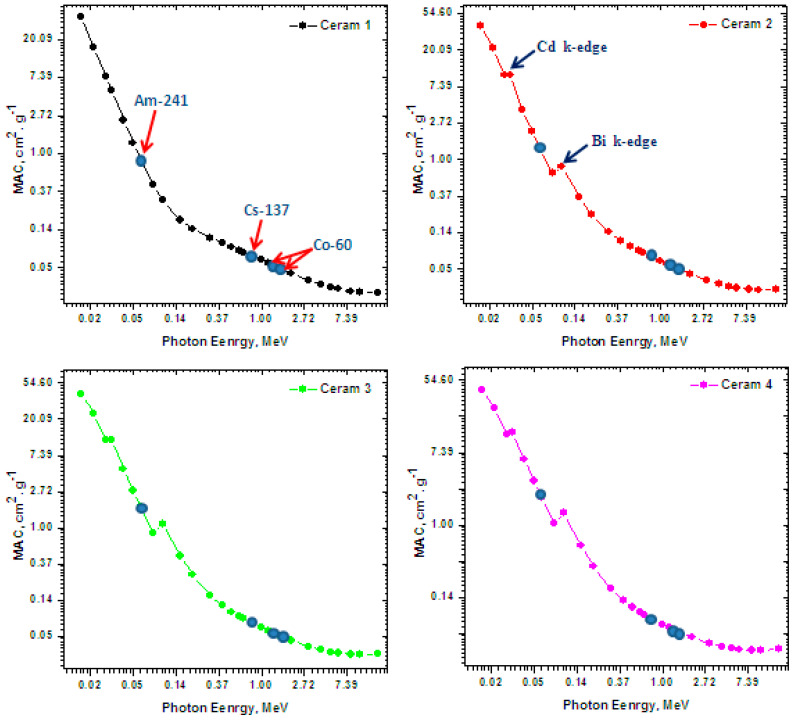
The mass attenuation coefficients (MAC) of the ceramic samples.

Figure 6 shows how the MAC of the ceramic samples varies according to energy from 0.015 MeV and 15 MeV. Additionally, the experimental values obtained from radioisotopes (Am-241, Cs-137, and Co-60) were added to the same figure (as blue circles) to demonstrate the MAC values at the energies emitted by these radioisotopes. The MAC for the four investigated ceramics decreased with increasing energy. For example, the MAC of Ceram 3 decreased from 40.28 cm^2^/g at 0.015 MeV to 11.22 cm^2^/g at 0.03 MeV, 0.081 cm^2^/g at 0.662 MeV, and 0.044 cm^2^/g at 2.00 MeV. This result indicates that the ceramics are good attenuators at low energy, and their radiation shielding ability decreases with increasing energy to low levels when trying to absorb very high-energy radiation. Additionally, Ceram 2–4 have two small peaks at two specific energies (marked in the subfigure for Ceram 2). Meanwhile, Ceram 1 does not contain any CdO or Bi_2_O_3_, which is why these peaks are not observed for this sample. When comparing the samples against each other, at every tested energy, Ceram 4 had a higher MAC than the other ceramic samples. At 0.03 MeV, the MAC values are equal to 5.280, 10.041, 11.328, and 12.814 cm^2^/g for Ceram 1–4, respectively, while at 0.20 MeV they are equal to 0.139, 0.224, 0.270, and 0.316 cm^2^/g for the same respective samples. These results indicate that ceramic 4 has the most desirable shielding ability.

**Figure 7:**
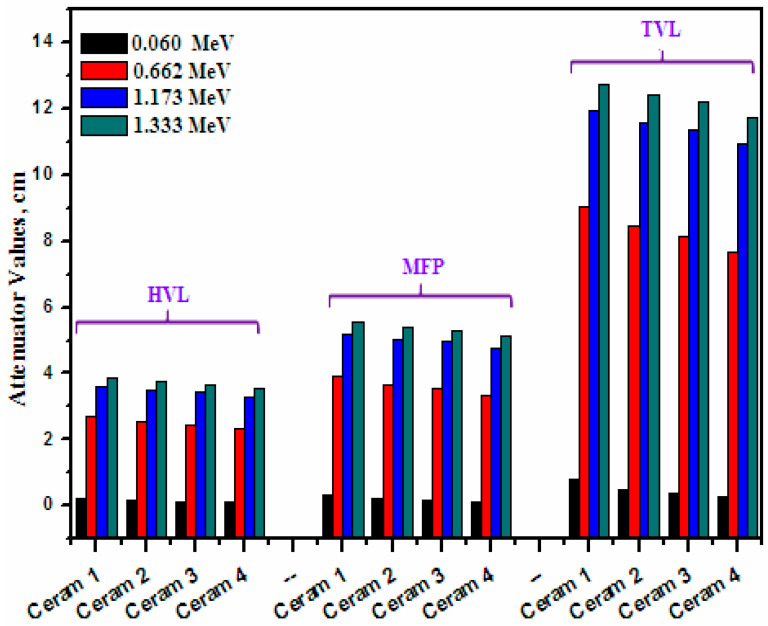
The HVL, MFP, and TVL for the prepared ceramics.

**Figure 8:**
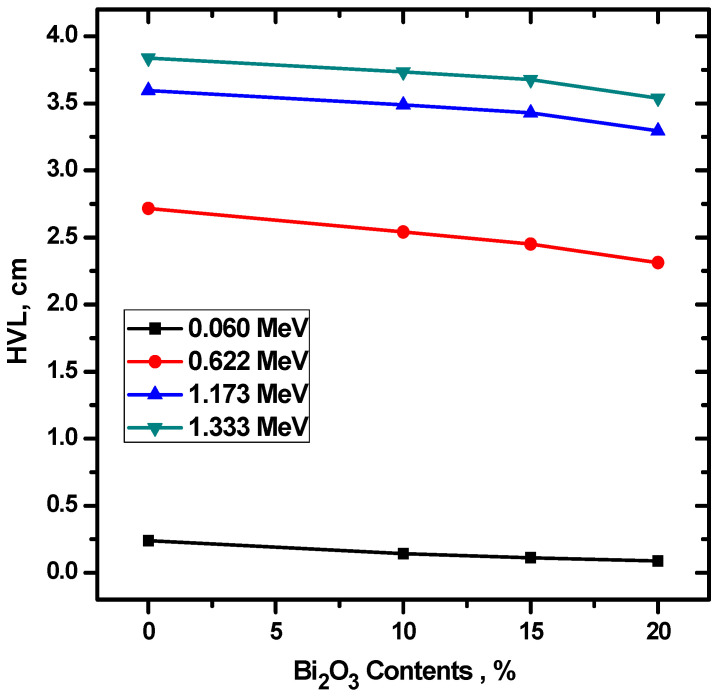
The HVL for the prepared ceramics as a function of Bi_2_O_3_.

**Figure 9:**
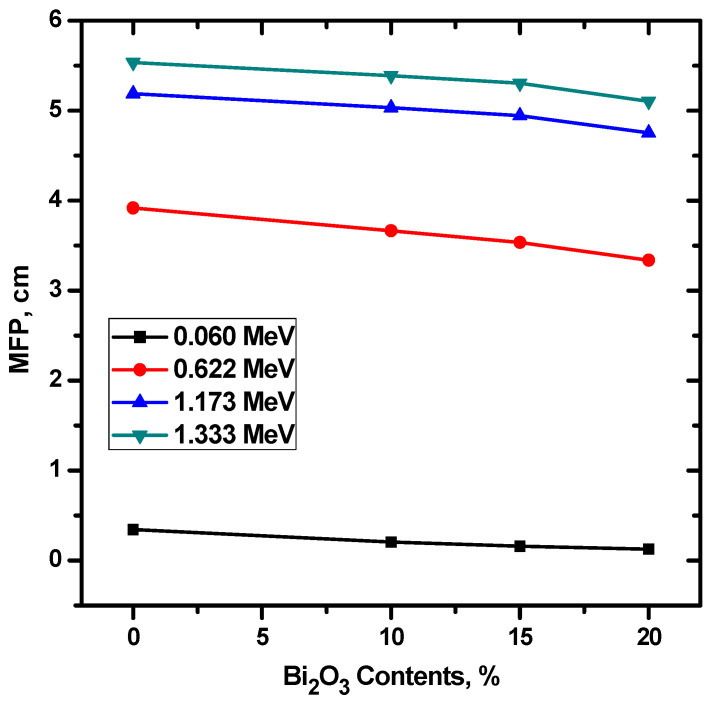
The MFP for the prepared ceramics as a function of Bi_2_O_3_.

The HVL, MFP, and TVL of the four investigated ceramics were experimentally calculated and the results were graphed in Figure 7. Ceram 4 has the lowest HVL, MFP, and In addition, there is a direct relationship between energy and the three parameters, meaning that they all increase with increasing energy, for all four ceramic samples. Since, by definition, TVL is the thickness of a material needed to reduce the intensity of the incoming photons by 90% of its original intensity, while HVL only looks at reducing the intensity to 50%, all the TVL values are much greater than the HVL values. For instance, at 0.0595 MeV, ceramic 3’s HVL is equal to 0.111 cm and its TVL is equal to 0.369 cm, while at 1.333 MeV, they are equal to 3.676 cm and 12.213 cm, respectively. In order to examine the influence of the chemical composition on the attenuation factors, we plotted the HVL as a function of Bi_2_O_3_ (Figure 8). It is to be noted that the HVL is decreased as the Bi2O3 increases, especially at low energy. This is due to the fact that, when the CuO is replaced by Bi_2_O_3_ in the composite materials, the density of the sample is enhanced, and it is known that the HVL is varies inversely with the density of the shield [26]. Therefore, adding more Bi_2_O_3_ to the prepared composite materials causes a reduction in the HVL as can be observed in Figure 8. The relation between the Bi2O3 and MFP is found for the same composite (see Figure 9).

**Figure 10:**
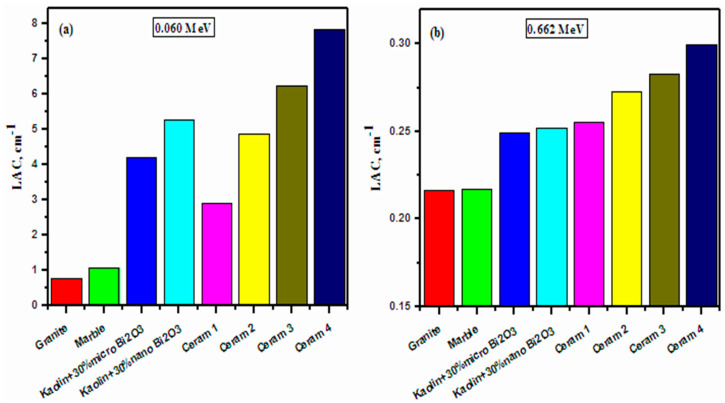
Comparison between the LAC of the prepared ceramics with other materials.

Figure 10 graphs the LAC of the four ceramic samples, as well as four other radiation shielding materials, at two chosen energies, 0.060 MeV and 0.662 MeV. The other four materials added for comparison are granite and marble [38], two other common materials, and Kaolin + 30% micro Bi_2_O_3_ and Kaolin + 30% nano Bi_2_O_3_ [39], which have been previously tested and proposed as shielding materials. At both energies, granite and marble have the lowest LAC values, while Ceram 2–4 have the greatest LAC values, with ceramic 4 having the greatest LAC value out of all the samples. More specifically, ceramic 4 has an LAC value of 7.823 cm^−1^ at 0.060 MeV and an LAC value of 0.300 cm^−1^ at 0.662 MeV. At 0.060 MeV, both the Kaolin-based shields have a higher LAC than Ceram 1, but at 0.662 MeV, Ceram 1 overtakes the two shields with an LAC of 0.255 cm^−1^. This figure reinforces the conclusion that the investigated ceramics are adequate materials to be used for radiation shielding purposes.

## 4. Conclusions

Gamma-ray shielding properties of Bentonite ceramic samples containing Copper oxide (CuO), Cadmium oxide (CdO), and Bismuth oxide (Bi_2_O_3_) have been studied at energy 0.06, 0.662, 1.173, and 1.333 MeV. Considering the value of LAC, it has been found that incorporating a bigger amount of CdO and Bi_2_O_3_ into Bentonite ceramics boosts their radiation shielding ability. However, at two special energies, 0.060 MeV and 0.662 MeV; the LAC values of prepared Bentonite ceramics have been compared with other earlier tested granite, marble, (Kaolin-30% micro Bi_2_O_3_), and (Kaolin-30% nano Bi_2_O_3_) materials. All the prepared Bentonite ceramics showed greater values than previously studied granite, marble, (Kaolin-30% micro Bi_2_O_3_), and (Kaolin-30% nano Bi_2_O_3_) materials. Finally, it can be concluded that prepared Bentonite ceramics are suitable as radiation shielding materials. Finally, the outcomes of this investigation show that the addition of CdO and Bi_2_O_3_ content in the Bentonite ceramics display better radiation shielding aptitude than pure Bentonite ceramics as well as granite and marble.

## Figures and Tables

**Table 1:** Chemical compositions of the bentonite clay used in this work.

Oxides	SiO_2_	Al_2_O_3_	CaO	Fe_2_O_3_	TiO_2_	Na_2_O	MgO	K_2_O	SO_3_	L.O.I
Percentage (%)	47.65	19.35	9.92	9.62	2.54	1.27	1.18	1.18	1.66	5.63

**Table 2:** Chemical compositions, densities, and codes of the present ceramic samples.

Sample Code	Compositions, wt (%)				Density (g/cm^3^)
	Bentonite	CuO	CdO	Bi_2_O_3_	
**Ceram 1**	50	50	-	-	3.398 ± 0.008
Ceram 2	50	35	5	10	3.465 ± 0.011
Ceram 3	50	25	10	15	3.509 ± 0.007
Ceram 4	50	15	15	20	3.634 ± 0.010

## Data Availability

Not applicable.
